# Innumerable cerebral microbleeds in hepatitis B virus-related decompensated liver cirrhosis: a case report

**DOI:** 10.1186/s12883-021-02291-9

**Published:** 2021-06-26

**Authors:** Chi Hyuk Oh, Jin San Lee

**Affiliations:** 1 Department of Internal Medicine, Kyung Hee University Hospital, Kyung Hee University College of Medicine, Seoul, 02447 South Korea; 2 Department of Neurology, Kyung Hee University Hospital, Kyung Hee University College of Medicine, #23 Kyunghee-daero, Dongdaemun-gu, Seoul, 02447 South Korea

**Keywords:** Cerebral microbleeds, Cerebral amyloid angiopathy, Hepatic encephalopathy, Chronic liver disease, Case report

## Abstract

**Background:**

Cerebral microbleeds (CMBs) are small, rounded, dark-signal lesions on brain MRI that represent cerebral hemosiderin deposits resulting from prior microhemorrhages and are neuroimaging biomarkers of cerebral amyloid angiopathy (CAA). Here, we report a case of innumerable CMBs in a patient with hepatic encephalopathy underlying decompensated liver cirrhosis.

**Case presentation:**

An 83-year-old woman diagnosed with hepatitis B virus-related liver cirrhosis 40 years before was referred to our neurology clinic for progressive disorientation of time and place, personality changes, and confusion with somnolence over 2 weeks. Based on the laboratory, neuroimaging, and electrophysiological findings, we diagnosed the patient with hepatic encephalopathy, and her symptoms recovered within 12 h after proper medical management. Brain MRI showed innumerable CMBs in the bilateral frontal, parietal, temporal, and occipital lobes. Since the distribution of CMBs in the patient was mainly corticosubcortical and predominantly in the posterior cortical regions, and the apolipoprotein E genotype was ε4/ε4, we speculated that CAA and hepatic encephalopathy coexisted in this patient.

**Conclusions:**

We suggest that severe liver dysfunction associated with long-term decompensated liver cirrhosis may be related to an increased number of CMBs in the brain. Our findings indicate that decompensated liver cirrhosis may be a risk factor for the development of CMBs and corroborate a link between the liver and the brain.

## Background

Cerebral microbleeds (CMBs) are small, rounded, dark-signal lesions on brain MRI that represent cerebral hemosiderin deposits that result from prior microhemorrhages and are neuroimaging biomarkers of cerebral small vessel disease, such as arteriosclerosis and cerebral amyloid angiopathy (CAA). Growing evidence suggests that CMBs are associated with an increased risk of hemorrhagic or ischemic stroke, cognitive decline, and dementia [1]. Recently, a few studies have demonstrated a positive correlation between the severity of chronic liver disease and the number of CMBs [2, 3]. We report a case of innumerable CMBs in a patient with hepatic encephalopathy underlying decompensated liver cirrhosis.

## Case presentation

An 83-year-old woman was referred to the neurology clinic for progressive disorientation of time and place, personality changes such as apathy and irritability, and a confusion with somnolence over 2 weeks. Forty years earlier, the patient was diagnosed with hepatitis B virus-related liver cirrhosis. After diagnosis, she was repeatedly hospitalized and managed for recurrent esophageal variceal bleeding, ascites, and hepatic encephalopathy. The patient’s daughter reported that the patient had developed progressive memory decline over 2 years; however, cognitive decline was not evaluated because of changes in her medical condition. She also had a history of diabetes, dilated cardiomyopathy, and atrial fibrillation. No familial history of stroke or dementia was noted. On neurological examination, the cranial nerves were intact; she had normal movements of the extraocular muscles. Motor examination revealed normal muscle strength and tone; however, she had diminished deep tendon reflexes, bradykinesia, and hypomimia. The Babinski sign was not elicited, and the sensory examination results were normal. Hepatic encephalopathy was suspected, and a laboratory and imaging diagnostic workup was performed.

**Fig. 1:**
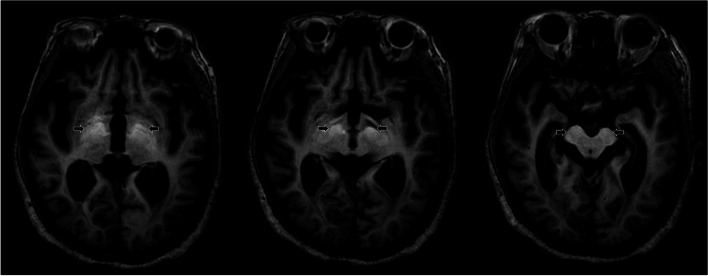
Brain MRI shows bilateral symmetric high signal intensity at the globus pallidus and substantia nigra on T1-weighted image (arrows)

**Fig. 2:**
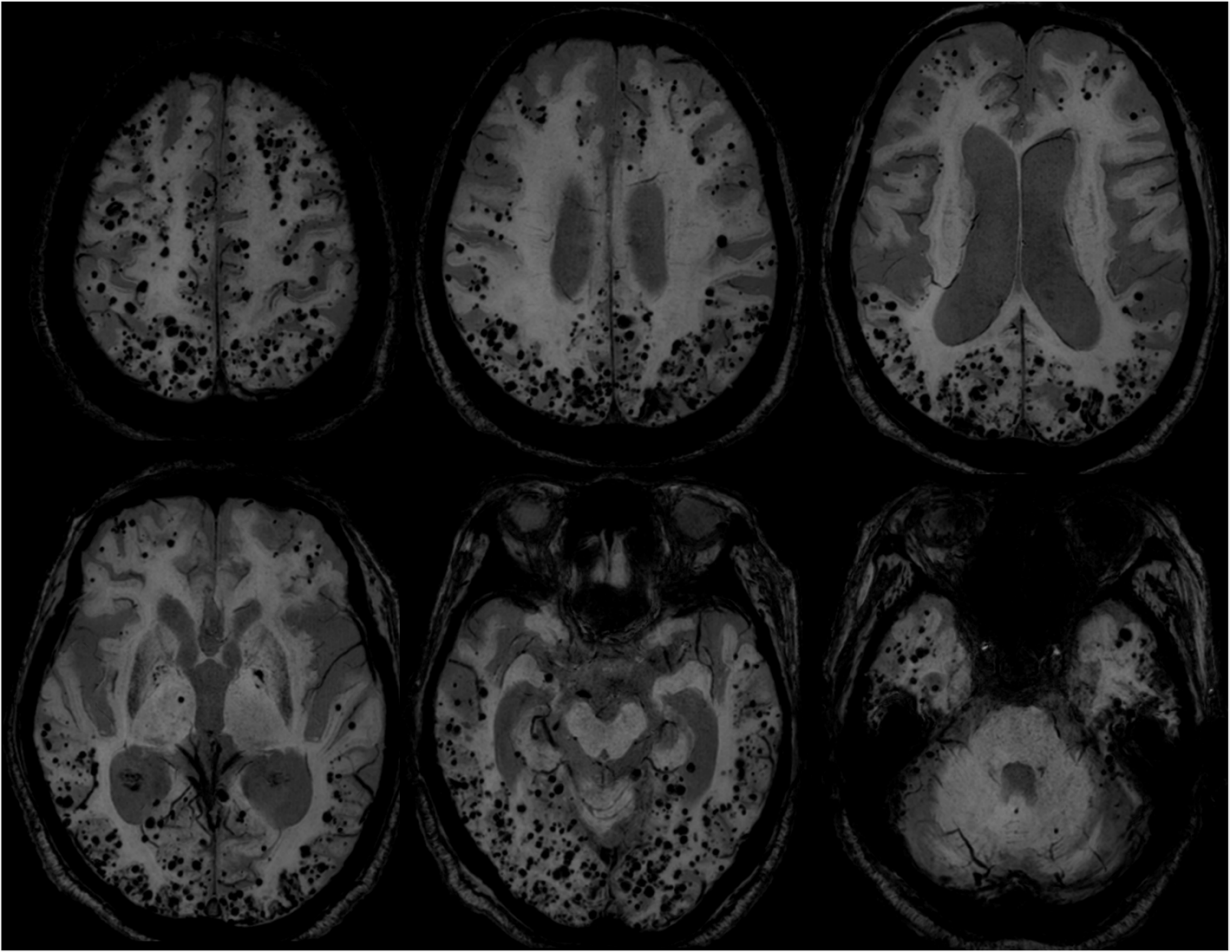
Innumerable cerebral microbleeds were observed at the bilateral frontal, parietal, temporal, and occipital lobes on susceptibility-weighted imaging sequence (dark dots)

The results of laboratory tests were as follows: total bilirubin, 1.95 mg/dL (0.3 ~ 1.2 mg/dL); albumin, 2.2 d/dL (3.5 ~ 5.2 d/dL); PT INR, 2.25 (0.9 ~ 1.2); ammonia, 349 ug/dL (27 ~ 90 μg/dL). The patient’s kidney function was within the normal range, and she did not have proteinuria or albuminuria. There were no symptoms or signs of systemic amyloidosis. Brain MRI revealed bilateral symmetric high signal intensity at the globus pallidus and substantia nigra on T1-weighted images (Fig. 1). In addition, electroencephalography showed triphasic waves and bursts of intermittent rhythmic delta activity. Considering the laboratory, neuroimaging, and electrophysiological findings, we diagnosed the patient with hepatic encephalopathy grade 3 based on the West Haven criteria. Treatment with lactulose enema and intravenous hydration with branched-chain amino acids was immediately initiated, and her symptoms recovered within 12 h. The patient scored 22/30, after losing 1, 4, and 3 points on the temporal orientation, attention and calculation, and recall of three items tasks, respectively, in the Mini-Mental State Examination. However, we found innumerable CMBs in the bilateral frontal, parietal, temporal, and occipital lobes on the susceptibility-weighted imaging sequence (Fig. 2). Since the distribution of CMBs in the patient were mainly corticosubcortical (grey-white matter junction) and predominantly in the posterior cortical regions, with no family history suggesting hereditary CAA, and the apolipoprotein E (*APOE*) genotype was ε4/ε4, we speculated that sporadic CAA and hepatic encephalopathy coexisted in this patient.

## Discussion and conclusions

CAA is a cerebrovascular disease caused by the accumulation of β-amyloid in the tunica media and adventitia of cortical and leptomeningeal small vessels [1]. The clinical presentation of CAA includes spontaneous lobar intracranial hemorrhage (ICH), cognitive impairment and dementia, and transient focal neurological episodes. The only known genetic risk factors for sporadic CAA are *APOE* alleles, the ɛ4 allele for CAA, and the ɛ2 allele for CAA-related ICH and hematoma expansion [1]. Definite diagnosis of CAA can only be made after a postmortem pathologic examination of the brain. However, multiple lobar CMBs on brain MRI have been successfully validated as a diagnostic marker for CAA, and 90% of neuroimaging-diagnosed CAA patients had moderate to severe CAA on neuropathology [1].

The CMBs in the present case were uncountable, and this unique neuroimaging finding has never been reported. To the best of our knowledge, the observed number of CMBs was a distinct feature of this patient, being greater than that in typical CAA [4, 5]. We suggest that, along with the patient’s *APOE* genotype, severe liver dysfunction associated with long-term decompensated liver cirrhosis may be related to an increased number of CMBs in the brain. Previous studies demonstrated that chronic liver diseases are associated with endothelial dysfunction, inflammation, and arterial stiffness in other organs, which are also related to the development of CMBs [2, 3]. In addition, we speculate that the severe liver dysfunction and underlying CAA in this case caused an increase in the number of CMBs through the breakdown of the blood–brain barrier (BBB), since increased BBB permeability is known to underlie hepatic encephalopathy [6]. However, we could not rule out the possibility that the patient’s impaired coagulation function contributed to the number of CMBs, since a greater variability in INR is associated with a higher prevalence of CMBs [7].

To our knowledge, this is the first neuroimaging report of a patient with innumerable CMBs and hepatic encephalopathy. Our findings indicate that decompensated liver cirrhosis may be a risk factor for the development of CMBs and corroborate a link between the liver and the brain. Further studies are needed to evaluate the pathophysiological mechanisms of CAA in patients with chronic liver disease.

## Data Availability

Data sharing is not applicable to this article, as no datasets were generated or analyzed during the current study.

## Abbreviations

CMBs: Cerebral microbleeds
CAA: Cerebral amyloid angiopathy
*APOE*: Apolipoprotein E
ICH: Intracranial hemorrhage
BBB: Blood–brain barrier
